# Tying malaria and microRNAs: from the biology to future diagnostic perspectives

**DOI:** 10.1186/s12936-016-1222-9

**Published:** 2016-03-15

**Authors:** Mercedes Rubio, Quique Bassat, Xavier Estivill, Alfredo Mayor

**Affiliations:** ISGlobal, Barcelona Ctr. Int. Health Res. (CRESIB), Hospital Clínic-Universitat de Barcelona, Carrer Rosselló 153 (CEK building), 08036 Barcelona, Spain; Centro de Investigação em Saúde da Manhiça (CISM), Maputo, Mozambique; Centre for Genomic Regulation (CRG), The Barcelona Institute of Science and Technology; CIBER in Epidemiology and Public Health (CIBERESP), Universitat Pompeu Fabra (UPF), Barcelona, Spain; Experimental Genetics, Sidra Medical and Research Centre, Doha, Qatar

**Keywords:** Malaria, MicroRNA, Disease, Severity, Biomarker, Diagnosis

## Abstract

Symptoms caused by bacterial, viral and malarial infections usually overlap and aetiologic diagnosis is difficult. Patient management in low-resource countries with limited laboratory services has been based predominantly on clinical evaluation and syndromic approaches. However, such clinical assessment has limited accuracy both for identifying the likely aetiological cause and for the early recognition of patients who will progress to serious or fatal disease. Plasma-detectable biomarkers that rapidly and accurately diagnose severe infectious diseases could reduce morbidity and decrease the unnecessary use of usually scarce therapeutic drugs. The discovery of microRNAs (miRNAs) has opened exciting new avenues to identify blood biomarkers of organ-specific injury. This review assesses current knowledge on the relationship between malaria disease and miRNAs, and evaluates how future research might lead to the use of these small molecules for identifying patients with severe malaria disease and facilitate treatment decisions.

## Background

The burden of infectious diseases disproportionately affects the African continent, where massive diagnostic needs have to be met with limited resources. Considerable advances have been made in the management of African patients with febrile illness and in ensuring that those who are severely ill can have access to hospital [1]. However, current evidence suggests that many of the existing guidelines are not followed [2], laboratory workload compromises quality, and prioritizing hospital admissions is rarely practised [3]. Moreover, access to the health system remains severely compromised for African patients, particularly in the most rural areas. Efforts are needed to improve diagnostic capacity for the clinical management of patients in this challenging environment.

Pneumonia and malaria are two leading causes of morbidity and mortality in children aged under 5 years worldwide [4], especially in malaria-endemic countries, where diagnostic tools are scarce and access to adequate care is limited [5, 6]. More than 60 % of the estimated 905,059 pneumonia deaths in 2013 among children <5 years occurred in 10 developing countries [7], and are mainly secondary to bacterial infections [8]. There were an estimated 214 million cases of malaria worldwide (range 149–303 million) in 2015 and 438,000 deaths (range 236,000–635,000), with 91 % of all malaria deaths occurring in sub-Saharan Africa and children aged under 5 years bearing the largest burden [9]. To reduce the number of deaths caused by pneumonia and malaria, early diagnosis is critical for a promptly administration of the adequate treatment.

An optimal management of malaria needs a correct diagnosis and a good assessment of severity. However, the distinction on clinical grounds between malaria and other infectious diseases, such as severe pneumonia, is not straightforward and has challenged clinicians for a long time [10–12]. Clinical signs of malaria in children are highly unspecific and often overlap with those of other infections, such as pneumonia, bacteraemia or meningitis [13–16]. In such a context, severe malaria is difficult to differentiate from other infections, which often include the appearance of respiratory signs and fever. Even when a *Plasmodium* infection is confirmed, causal attribution of symptomatology to malaria is not straightforward, as malaria-positive slides are a common incidental finding in areas where many people are semi-immune to malaria and have high rates of asymptomatic parasite carriage [17]. Thus, the detection of parasites in the blood of sick patients is not a definitive proof of their association with the clinical symptoms.

The importance of this disease-overlap will depend upon the epidemiology of pathogens in each particular setting, but also on the severity of the presentation and the age of the patient. One consequence of falling malaria transmission rates [18, 19] is that presumptive treatment is not as safe as it was a decade ago. This is because the malaria-attributable fraction of fevers is now substantially lower and therefore the relative likelihood of failing to treat alternative causes of severe infection has become higher [20], potentially leading to avoidable morbidity and mortality. Moreover, overdiagnosis of malaria in endemic regions increases the use of anti-malarial drugs which contributes to the development of anti-malarial drug resistance [21], and also burdens health services with unaffordable costs [22]. Additionally, diagnosis is hindered by the broad diversity of pathophysiological pathways, leading to the different syndromic presentations associated with severe malaria [23]. Current World Health Organization recommendations state that it is essential to give promptly full doses of effective parenteral (or rectal) antimalarial treatment in the initial treatment of severe malaria, followed by a full dose of effective artemisinin-based combination therapy orally [24]. The identification at early infection stages of those individuals at risk of severity and death can help to save lives by providing a prompt aggressive treatment. Biomarkers capable to predict the likelihood of complications both at initial presentation and during antimalarial and supportive treatment could be used to guide the management of patients most at risk of severe pathologies. Finally, the diagnostic revolution that the introduction of rapid diagnostic tests (RDTs) has meant to the diagnosis of malaria has also had the counter-effect of leaving clinicians with a significant amount of non-malarial fevers with no diagnostic filiation.

The diversity of the causes of fever, most of which cannot be diagnosed on clinical grounds alone, calls for the development of point-of-care tests to make rapid clinical decisions in developing countries. Any improvement on clinical endpoints to differentiate malaria from other infectious diseases with no requirement of laboratory facilities might help management of treatment. Further improvement could be brought by integrating host biomarkers able to predict children at risk of dying. Several efforts have been made in recent years to evaluate multiple host biomarkers in the differential diagnosis of malaria, bacterial or viral infections, but to date no single satisfying biomarker has shown a clinical utility to reliably distinguish these three microbial aetiologies [6, 25, 26]. A new area of research that holds great potential for individualized medicine is the study of small non-coding RNAs. Since the discovery of microRNAs (miRNAs) in 2001 [27], numerous studies have attempted to evaluate them as promising biomarkers for several human disorders such as cancer, cardiovascular diseases, diabetes, exposure to toxic compounds, and infectious diseases [28, 29]. This is so because dysregulation of the expression of these tiny molecules, which control in an extremely accurate way metabolic processes such as apoptosis, cell proliferation and differentiation [30], is usually indicative of pathological disorders or physiological changes in the individual. For that reason many studies in the last few years have tried to correlate aberrant miRNA levels with specific disease in order to find new biomarkers and diagnosis tools. This review aims to describe the current knowledge about miRNAs in malaria and discuss their potential as biomarkers of pathologies associated with this infectious disease.

## Malaria disease

In 2015 malaria was endemic in 97 countries, with 3.2 billion people, almost half of global population, living at risk of infection. This scourge is responsible for over 200 million annual cases, and nearly half a million annual deaths every year [31]. Malaria can be caused by five species of *Plasmodium: Plasmodium falciparum, P. vivax*, *P. malariae, P. ovale,* and *P. knowlesi. Plasmodium* is an apicomplexan parasite with a complex life cycle involving two different hosts: mosquitoes as vectors and vertebrates as the final host. Anopheline mosquitoes inject malaria parasites (sporozoites) into the subcutaneous tissue of the vertebrate host. Sporozoites travel to the liver and infect the hepatocytes, where each one develops into thousands of merozoites. In the blood, merozoites grow inside red cells through successive cycles of invasion, asexual replication and release of newly formed merozoites. Some of these merozoites will be developed into sexual forms (gametocytes) which will be taken up by other mosquitoes when they feed from infected blood [32]. In the case of *P. vivax* and *P. ovale*, parasites may persist in the liver as dormant stages called hypnozoites, eventually leading to relapses weeks, months and years after a primary infection [33].

All the clinical symptoms associated with malaria are caused by the asexual erythrocytic or blood-stage parasites. The rupture of *Plasmodium* schizont stage-infected erythrocytes releases merozoites into the bloodstream [34], as well as parasite-derived toxins such as haemozoin pigment, glycosyl-phosphatidyl-inositol molecules that anchor a diverse range of proteins to the surface of malaria parasites, and other by-products of the parasite. These toxins stimulate macrophages and other innate immune cells to produce pyrogenic-inflammatory mediators and cytokines that stimulate thermoregulatory regions of the brain to increase body temperature, leading to the acute periodic febrile episodes [35]. Although most of the malaria infections are associated with uncomplicated malaria, a percentage of them evolve into severe disease, especially those infections caused by *P. falciparum*. Severe malaria in African children encompasses at least three main clinical syndromes that can occur alone or in combination: severe malarial anaemia, cerebral malaria and respiratory distress (often secondary to metabolic acidosis [36]). Symptoms of severe malaria are similar to those of many other diseases that are common in malaria-endemic countries, such as septicaemia, severe pneumonia and typhoid fever [37].

**Fig. 1:**
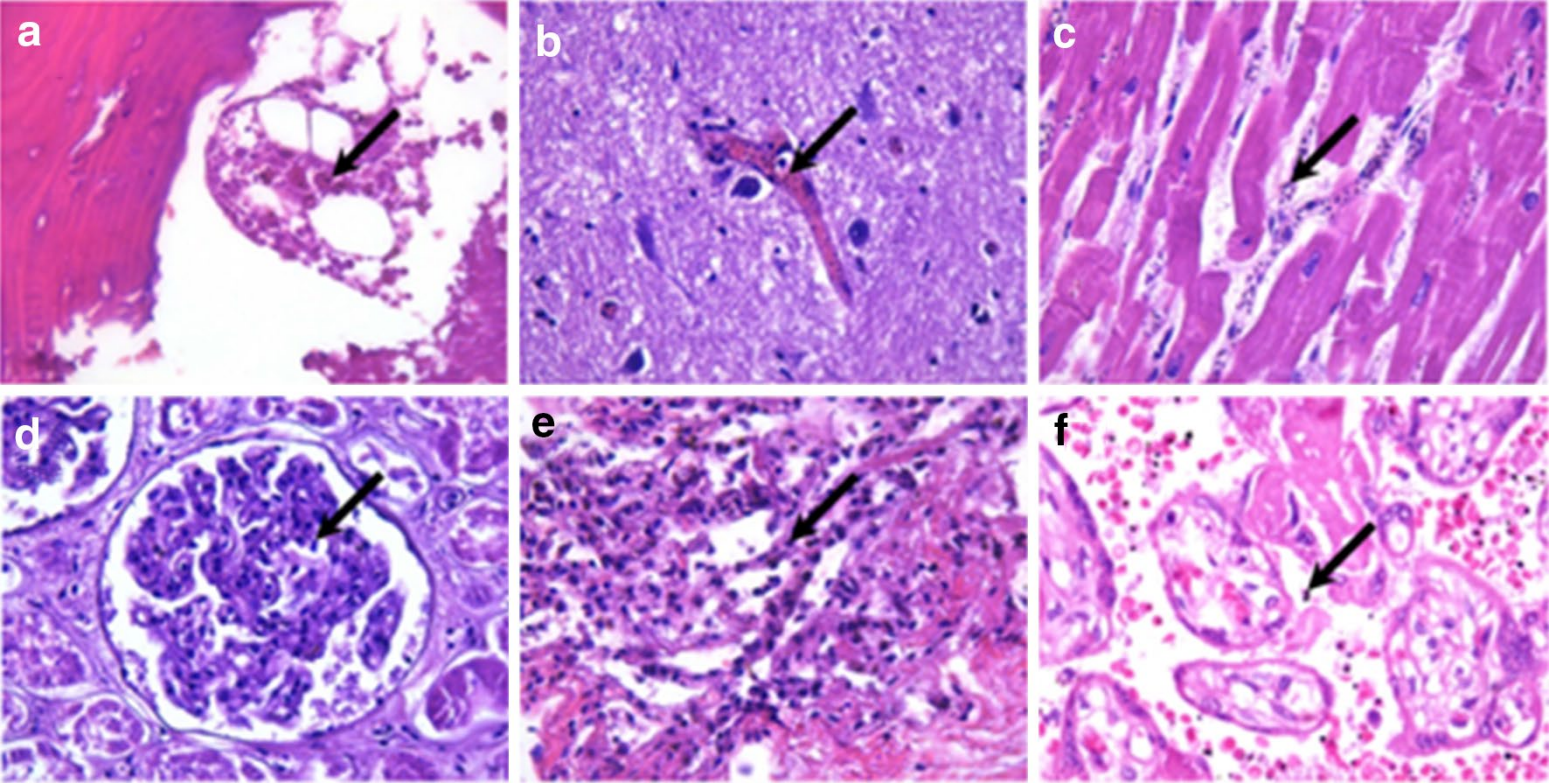
Sequestration of *Plasmodium falciparum*-infected erythrocytes in the capillaries of different human tissues. Tissue specimens for histological analysis were fixed in 10 % neutral buffered formalin-paraffin embedded and stained with haematoxylin and eosin (H and E). **a** Bone marrow (H and E, 400x), **b** Central nervous system (H and E, 400x), **c** Heart (H and E, 400x), **d** Kidney (H and E, 400x), **e** Lung (H and E, 400x), **f** Placenta (H and E, 400x). *Arrows* indicate infected erythrocytes (*blue*). Photographs by Paola Castillo and Jaume Ordi

In terms of the underlying pathophysiology, these severe forms of malaria infection, mainly characteristic of *P. falciparum,* have been attributed to the ability of erythrocytes infected by this species to sequester in vital organs of the human host such as brain [38], placenta [39] and bone marrow [40] (Fig. 1). Erythrocytes infected by mature stages of the parasite adhere to cellular endothelium through interactions with human receptors such ICAM-1, CD36, gC1qR endothelial protein C receptor (EPCR), to non-infected erythrocytes forming ‘rosettes’ or to platelets to form agglutinates [41, 42]. Although there is still some debate, severe malaria has been associated with some of these cyto-adhesive phenotypes [42, 43] and the subsequent microvascular obstruction resulting from the accumulation of infected erythrocytes in vital organs [44]. The parasite protein expressed on the surface of infected erythrocytes that mediates this cyto-adhesion has been identified as a very large protein called *P. falciparum* erythrocyte membrane protein 1 (PfEMP1), encoded by approximately 60 *var* genes per genome [45]. Sequestration of *P. falciparum* in the microvasculature is thought to avoid clearance of the parasite in the spleen. However, such accumulation of parasites provokes increasing vascular resistance, inflammatory reactions and tissue damage [46]. The ensuing distal tissue hypoxia can increase host anaerobic glycolysis due to a mismatch between tissue oxygen supply and requirement [47, 48], contributing to malarial acidosis [48]. Moreover, binding of PfEMP1 to host receptors such as a EPCR can also contribute to intensive inflammation through the blocking of cytoprotective functions mediated by the activated protein C (APC) cellular pathway [43]. However, not all the sequestration events may be exclusively mediated by cyto-adhesion of the parasite. Decreased deformability of erythrocytes infected by immature gametocyte stages [49] may play a role in their enrichment in the bone marrow [40, 50], potentially disturbing haematopoietic functions of this organ. Moreover, severe malaria can also result also from an excessive host inflammatory response following exposure to malaria ‘toxins’ [51] or metabolic damage of critical organs [23]. These mechanisms may explain severe malaria syndromes such as lactic acidosis that result from a combination of anaerobic glycolysis in tissues where sequestered parasites interfere with microcirculatory flow, lactate production by the malaria parasites, and a failure of hepatic and renal lactate clearance [23].

## Malaria diagnostics

Malaria case management, which consists of prompt diagnosis and effective treatment, remains a vital component of malaria control and elimination strategies [24]. High-quality malaria diagnosis is important in all settings as misdiagnosis can result in significant morbidity and mortality [14]. Recent experience in malaria case management has shown that using appropriate diagnostic tools such as malaria RDTs has the potential to improve rational use of anti-malarial drugs [52]. However, it has often been accompanied with an increased antibiotic prescription that is unlikely to provide clinical benefit in most cases [53], but rather exposes the patient to unnecessary adverse events and increases the development of antimicrobial resistance.

Malaria is classically identified by optical microscopic examination of Giemsa-stained peripheral blood smears. Although microscopy is easy to perform and cost effective, the technique is time consuming and requires maintaining the proficiency of microscopists through extensive training and quality-control procedures. Theoretically, thick film microscopy by highly skilled technicians can detect approximately 20–50 parasites/µL, with detection limit of 100–500 parasites/µL in most field settings [54]. However, the fact that *P. falciparum* can sequester in vital organs by cyto-adhesion to certain host receptors, complicates the diagnosis as parasites might be present in tissues, such as the placenta in pregnant women or the brain, but absent in peripheral blood [55].

RDTs employ immunochromatographic methods to detect parasite proteins [*P. falciparum* histidine-rich protein 2 (HRP2)] or glycolytic enzymes (*Plasmodium* lactate dehydrogenase and aldolase) with a working threshold of approximately 100 parasites/µL [56], although more sensitive RDTs are currently under development. RDTs do not require electricity or a water source, are simple to use, easy-to-interpret and relatively cost effective. The quantification in plasma of HRP2 has been shown to allow the estimation of total body parasite biomass with acute *P*. *falciparum* malaria [57], suggesting that detection of HRP2 may identify parasites sequestered away from peripheral blood [58]. However, HRP2-based RDTs can remain positive even after an infection has been successfully treated, as the antigen remains in circulation for days to even several weeks after parasitaemia has cleared, something that hampers their utilization for fevers occurring short-term after a malaria episode [59]. Moreover, parasites lacking the *hrp2* gene are beginning to emerge, possibly because they avoid detection by HRP2-based tests [60].

Polymerase chain reaction (PCR) is now well recognized as the most sensitive method to detect malaria parasites. Primers targeting the *18S ribosomal* gene specific for any *Plasmodium* species are most commonly used. Nested PCR [61] was the standard method until recently, when real-time PCR methods have become increasingly more common [62, 63]. Different PCR methods have limits of detection ranging from 2 to 2000 parasites/µL [64, 65]. Being the most sensitive and accurate diagnostic tool, its wider implementation in malaria-endemic countries appears challenging, because it needs qualified personnel, expensive infrastructure and reagents, as well as certain storage conditions of reagents. Other techniques that are being developed for the diagnostic of malaria parasites include loop-mediated isothermal amplification (LAMP), a DNA amplification method in which specific genes are made into loops so that DNA amplification can occur continuously [66], RNA hybridization assays [67], laser desorption mass spectrometry (LDMS) which detects haem from haemozoin as a marker of malaria infection [68], and flow cytometry and automated blood counters which detect haemozoin-containing leukocytes [69].

These diagnostic techniques still have limitations to detect some parasite stages and discriminate between disease states. Hypnozoites from *P. vivax* and *P. ovale* cannot be detected with currently available diagnostic tools and are not cleared upon treatment with routinely administered anti-malarial drugs, unless primaquine (a drug that requires at least seven to 14 days of administration and can cause severe haemolysis in people with glucose-6-phosphate dehydrogenase deficiency) is added to the treatment [70]. A tool to detect such dormant forms would be extremely useful for current elimination efforts, as hypnozoites constitute a parasite reservoir that can cause blood-stage infections at a later point in time [71]. Also, most common diagnostic tools cannot discriminate between severe or uncomplicated malaria. Current diagnosis tools for the detection of malaria, benefits and challenges are summarized in Table 1. However, other approaches have been assessed for the diagnosis of life-threatening malaria. Ophthalmoscopy has helped to increase accuracy of severe malaria diagnosis by allowing the visualization in real time of certain signs believed to be pathognomonic for the diagnosis of cerebral malaria, such as retinal whitening, papilloedema, vessel colour changes, and white-centred haemorrhages [72]. Any parameter associated with malaria-attributable severe disease could help to improve severe malaria diagnosis.

**Table 1:** Current diagnosis tools for the detection of malaria, benefits and challenges

Method	Detection	Benefits	Limitations
Clinical diagnosis	Observation of symptoms	Fast and inexpensive	Cannot detect asymptomatic malaria
			Some symptoms overlap with other diseases
Giemsa microscopy	Detection of parasites in blood smears	Fast and inexpensive	Requires trained personnel
		Able to differentiate malaria species	Requires maintenance of infrastructures
		Able to quantify parasites	Need to establish good quality controls and quality assurances
			Cannot detect sub-microscopic infections
Rapid diagnosis test (RDT)	Detection of malaria antigens and enzymes in blood	Fast (5–20 min)	Variable sensitivity
		Simple and easy to interpret	Cross-reactivity with other blood factors
		No electricity needed	Affected by environmental conditions
			Cost effectiveness vary with malaria prevalence
			Cannot detect sub-RDT infections
Polymerase chain reaction (PCR)	Detection of parasite DNA by PCR	High sensitivity and specificity	Cost and technical limitations makes it not applicable in daily clinical work
		Determination of mixed infections	
		Able to quantify parasites	

## New biomarkers in malaria disease

Several molecules have been proposed to discriminate the aetiology of severe infections. Biomarkers to differentiate viral from bacterial infections have been evaluated mostly in the developed world to support clinical diagnosis and to be applied as RDTs. Despite disparity in results [73, 74], procalcitonin (PCT) and C-reactive protein (CRP) are routinely used in many emergency departments and intensive care units of developed countries as a rapid screening method to guide the aetiological diagnosis in children with pneumonia, assuming higher levels of both markers in bacterial infections when compared to viral ones. However, distribution between clinical groups has been shown to overlap in presence of malaria parasites [75], suggesting that malaria infection should be taken into consideration if using PCT or CRP to differentiate viral from invasive bacterial pneumonia in malaria-endemic areas. Haptoglobin in combination with lipocalin 2 has also been shown to allow discrimination between pneumonia and malaria in children with respiratory distress [26], and alone or in combination with tumour necrosis factor receptor 2 (TNFR2) or interleukin-10 (IL10), and tissue inhibitor of metalloproteinases 1 (TIMP1) has recently been suggested to allow accurate classification of patients into bacterial, malarial and viral aetiologies [25]. Erythropoietin (EPO), a glycoprotein hormone principally produced by the kidney’s peritubular capillary endothelial cells in response to hypoxia, has been shown to be increased in African children with malaria [76–80], suggesting that EPO might be a good indicator of malaria-attributable disease. However, the utility of EPO to distinguish malaria-attributable severe disease has been questioned due to the overlap of EPO values between children with severe malaria and invasive bacterial infection [81]. Similar limitations have been described for serum lactate/pyruvate [47, 48], with no differences found when comparing children with severe malaria and invasive bacterial infection [81].

Potential serological markers for cerebral malaria have been also suggested. Angiopoietin factors, which regulate the maintenance of vascular integrity, have been shown to be differentially regulated in patients with cerebral malaria. Compared to healthy controls, cerebral malaria patients have been shown to have reduced levels of angiopoietin 1 (ANG1), elevated levels of angiopoietin 2 (ANG2) and higher ANG2/1 ratios [82]; moreover, low ANG1 levels at presentation predicted subsequent mortality in children with cerebral malaria. Also, increases of a combination of multiple host markers of inflammation and endothelial activation, such as ICAM-1, sTie-2 and sFlt-1, have been observed in cerebral and severe malaria compared to uncomplicated malaria as being able to predict mortality [83]. Finally, levels of circulating HRP2 have been shown to predict parasite sequestration in tissues [57]. Moreover, several studies have explored the utility of HRP2 concentrations as a risk factor for mortality in severe malaria [57, 84–86]. Although concentrations were found to be higher in patients dying with severe malaria, the U-shaped curve describing the relationship between HRP2 and mortality risk [84] suggested a limited applicability of quantitative HRP2 as a prognosis marker. From the side of the host, several biomarkers [PCT, CRP, ICAM, ANG2, tumour necrosis factor-α (TNF-α), IFN-gamma-inducible protein 10 (IP-10)] have demonstrated a consistent statistical association with mortality in patients with severe or cerebral malaria in two or more studies [83, 85, 87]. All these molecules have been suggested as promising clinically informative biomarkers for severe malaria, although additional studies are still needed to address their utility as prognostic biomarkers and potential therapeutic targets in severe malaria. Other molecules such as miRNAs are currently being evaluated as indicators of malaria disease stages and progression, as well as the risk of death.

## miRNA structure and function

miRNAs are small, endogenous, non-coding RNAs of about 21–23 nucleotides that regulate gene expression at a post-transcriptional level. Together with the small interfering RNAs (siRNA), miRNAs are one of the two types of small RNA molecules central to the gene silencing pathway called RNA interference (RNAi). They incorporate into the RNA-induced silencing complex (RISC), and bind to 3′ untranslated regions (UTR) or 5′UTR of the messenger RNAs (mRNAs) with imperfect complementarity blocking translation of the target mRNA. Since the discovery in 2001 of the first miRNAs lin-4 and let-7 in *Caenorabditis elegans* [27], 35,828 mature miRNAs have been found in 223 species of animals, plants, fungi, protozoa, and viruses (miRbase, version 21 [88]). MiRNAs regulate different processes in animals, such as developmental timing, patterning and embryogenesis, differentiation and organogenesis, growth control, and programmed cell death or differentiation of stem cell lineages [89]. miRNAs also have an important role in modulating innate immune responses, such as differentiation, survival and function of immune cells, cytokine responses and intracellular signalling pathways [90].

miRNAs undergo molecular processing before becoming mature. Most of them are transcribed in the nucleus as long primary transcripts by RNA polymerase II. These primary transcripts can have more than one miRNA folded in hairpin structures called precursor miRNAs (pre-miRNAs) that are released from the primary transcript when they are cut by the ribonuclease Drosha (a RNase III). These pre-miRNAs are transported to the cytoplasm by the enzyme exportin 5 and recognized by another RNase III (Dicer) that cleaves the hairpin to produce a miRNA duplex. One of the strands of the duplex is incorporated into the RISC combined with the protein argonaute, which has endonuclease activity directed against mRNA strands that display extensive complementarity to their bound miRNA. The miRNA then guides the argonaute-RISC to the target mRNA, binds to the imperfectly paired miRNA sites located within it, and blocks transcript translation, eventually leading to mRNA degradation [30].

miRNAs can easily get into the circulation from specific cells and tissues through transportation in exosomes and microvesicles or associated to lipoproteins [high-density lipoproteins (HDL) and low-density proteins (LDL)] or protein complexes, including argonaute 2 [91]. The mechanisms by which miRNAs are actively exported from cells and packaged into appropriate carriers are currently poorly understood, but the evidence shows that some miRNAs are selectively exported from certain type of cells [92]. There are specific signalling pathways that regulate miRNA release [93] and each biological fluid has a distinct miRNA signature that is consistent among different individuals [94]. Despite the high amount of RNases in the body fluids, circulating miRNA are stable due to the protection they get through the vesicles or binding proteins, and are internalized into recipient cells, retaining their ability to recognize and repress mRNA targets [95].

## Circulating miRNAs as biomarkers of disease

A good biomarker has to be specific, with high sensitivity and able to make accurate, reliable and reproducible diagnostic predictions. Also, they have to be robust and as little invasive as possible. miRNAs have been shown to be rapidly released from tissues into the circulation with the development of pathology, and to reflect disease status as well as organ damage and injury [96]. As secreted miRNAs can be detected in biological fluids such as plasma [97] and are stable even after long and severe storage conditions, they are currently being explored as promising non-invasive biomarkers to assess and monitor the body’s pathophysiological status [98]. For this reason, there have been huge efforts during the last years to profile miRNAs in serum, plasma, urine, and other biofluids, and relate them to different diseases.

There are more than 1000 studies about miRNAs as biomarkers in human diseases, most of them in different types of cancer. Carcinogen cells actively secrete specific miRNAs into the circulation, sometimes supporting the growth and progression of the tumour [99]. Given that the secretion of these tumour-specific miRNAs can be found in different stages of the disease, their detection may be useful for early diagnosis and prognosis. However, circulating miRNAs can also be biomarkers of other diseases, such as inflammatory and neuropathic pain conditions [100], neurologic pathologies [101], endocrine [102], and cardiovascular disorders [103]. Although the information about circulating miRNAs as biomarkers in different diseases is abundant and increases on a daily basis, partly thanks to new advances in next-generation sequencing and the development of new arrays and miRNA profiling methods, commonly reported miRNA biomarkers have been found associated with a wide range of conditions and outcomes. Moreover, findings have not been reproduced in all the studies, pointing to the need for standardized sampling and processing protocols in order to identify reliable biomarkers [104].

The existence of tissue-specific miRNA signatures has been suggested in several studies [105–108], reinforcing the concept that circulating miRNAs can be informative of the tissue where pathology is occurring. Tissue-specific miRNA signatures have been described in different cell types [105, 106]. Specificity of circulating miRNAs from different injured organs has also been show in rats [107], with increases of miRNA concentrations in plasma associated with injuries in liver, muscle or brain. Human miRNA profiles have been shown to reflect the developmental lineage and differentiation state of the tumours, allowing the classification of different types of cancer [108]. Finally, a database (http://bioeng.swjtu.edu.cn/TSmiR) has been developed to provide information on interaction maps of transcription factors and tissue specific miRNAs from experimentally validated and predicted data [109]. miRNAs also have the potential of being informative of pathological processes associated with infections. When bacteria, viruses or other pathogens enter in the organism, several hundred host genes are altered and miRNAs act to clear the pathogen and at the same time to avoid consequences of dysregulated gene expression [90]. There are some studies about aberrant miRNA expressions during viral infections, including hepatitis B virus [110], hepatitis C virus [111], human immunodeficiency virus [112], Epstein Barr virus [113], bacterial pneumonia, and tuberculosis [114, 115] as well as some parasitic infections [116]. In filarial infections, parasite-derived miRNAs has been detected in the circulation of the host [117] and some nematode parasites can release exosomes with small RNAs to regulate innate immunity [118]. Few miRNAs have been found to be prominently expressed in mice infected by the apicomplexa *Toxoplasma gondii* [119]. Specificity of miRNAs has been show in Toxoplasmosis experimental infections compared to mice infected with *P. berghei*, *P. yoelii*, *P. chabaudi*, *Cryptosporidium parvum*, Mouse hepatitis virus, and *Staphylococcus aureus* [119], or in different types of lung affections, lung cancer, pulmonary tuberculosis, and pneumonia [115], but very little data is available on the differential host miRNA expression associated with different types of infections, as most studies identify dysregulated miRNAs compared to healthy controls.

## miRNAs and malaria infection

Although it is known that miRNAs regulate the immune system [120] and may play a major role in the regulation of host responses to *Plasmodium* infection, the current knowledge about the role of miRNAs in malaria disease is limited.

### Small RNAs in *Plasmodium*, anopheline vectors and host erythrocytes

*Plasmodium* species lack the canonical RNAi machinery of other eukaryotes. No dicer or argonaute proteins have been found in any of the ten species of *Plasmodium* with sequenced genome available, and exhaustive analyses using RNA-based gene silencing approaches have revealed that *Plasmodium* lacks the enzymology required for RNAi-based ablation of gene expression [121]. The absence of RNAi machinery in *Plasmodium* is not surprising assuming that RNAi could initially have evolved as a host defence mechanism against viruses and transposons. *Plasmodium* is not affected by viruses, and some authors consider that the loss of RNAi machinery in some parasites may provide them with an advantage to develop new strategies to escape host defences [122, 123]. Although there is no canonical mechanism for RNAi in *Plasmodium*, several studies have shown successful down-regulation of gene expression using dsRNA, suggesting the existence of non-canonical RNAi pathway, albeit not found until now [124–126].

To complete the complex life cycle of *Plasmodium,* gametocytes of the parasite have to be taken from the blood of infected vertebrate hosts by female *Anopheles* mosquitoes*. Plasmodium* parasites then invade the midgut of the mosquito, a critical step for the parasite because the host tries to eliminate the infection. The knockdown of mosquito dicer and argonaute 1 has been shown to lead to increased sensitivity to *Plasmodium* infection [127], suggesting that impairment of miRNA maturation in the mosquito may alter translational regulation of a variety of target genes that affect the parasite development in the invertebrate host. However, some studies have shown that the dysregulation of miRNA expression during *Plasmodium* infection in the mosquito is dependent on the stages along parasite progression [128, 129].

All miRNAs isolated from malaria-infected erythrocytes have been confirmed to be of human origin, suggesting there are no miRNAs of *Plasmodium* [130, 131]. This is in contrast to the large number of miRNAs that are expressed by the closely related protozoan *Toxoplasma* to regulate host gene expression [132]. However, it has been suggested that host miRNAs present in human erythrocytes may translocate into the parasite, some of them forming a chimera with *Plasmodium* mRNAs to block their translation [133]. In particular, the translocation of miR-451, a very abundant miRNA in sickle erythrocytes [134] and its integration into essential parasite messenger RNAs, has been shown to result in translational inhibition via impaired ribosomal loading. Such a mechanism was suggested to represent a unique host defence strategy against complex eukaryotic pathogens and may contribute to the cell-intrinsic resistance to malaria characteristic of sickle cell anaemia. To our knowledge, this process was only observed in in vitro analyses, with no further replication of this observation in biological samples collected from naturally exposed human populations, leaving many questions unresolved, such as the mechanism by which the miRNAs enter in *Plasmodium* and recognize target mRNAs, if it only happens in sickle cells or in all erythrocytes, and whether these miRNAs from the erythrocyte can get into the circulation and attack surrounding infected cells. Other small regulatory RNAs recently found in malaria parasites, such as circular RNAs (circRNAs) [135], which have been suggested to act as miRNA sponges [136], may also be involved in the regulation against the defences of the host.

### miRNA defence against *Plasmodium* in vertebrate hosts

Only four studies have looked for abnormal regulation of host miRNAs after a *Plasmodium* infection in rodent models. Reprogramming of host miRNA expression was observed in the female mouse liver after a primary infection with *P. chabaudi*. These changes persisted during re-infection in mice that have acquired some level of anti-malarial immunity, suggesting that some miRNAs may regulate the development of protective immunity against malarial blood stages of *P. chabaudi* [137]. Nevertheless, it was not possible to correlate the changes in expression of these miRNAs with specific immune responses. A second study showed miRNA differences between *P. berghei* infected mice with cerebral and non-cerebral malaria. Alterations of certain miRNAs coincided with increased levels of circulating inflammatory cytokines and apoptosis within the brain [138]. A third study found that most miRNAs species were downregulated in blood and liver after *P. chabaudi* infection. Although authors suggested that alterations of miRNAs allowed the upregulation of numerous target genes in response to infection, causal relationship between altered miRNA levels and pathology cannot be established [139]. Finally, mice injected with genetically attenuated parasites (GAP), which arrest in the liver and induce sterile immunity, were observed to have a significant increase of miR-155 in their livers, especially pronounced in non-parenchymal cells, including liver-resident macrophages (Kupffer cells) [140]. More importantly, the GAP’s protective capacity was improved with the administration of miR-155 using adeno-associated virus8 (AAV8), opening the path to the future use of miRNAs as malaria treatment.

Very little is known about the potential of plasma miRNAs as markers of *Plasmodium* infections. A study compared the level of few miRNAs detected by quantitative PCR in the plasma of 19 individuals infected with malaria (three *P. falciparum* infections and 16 *P. vivax* infections) with levels of 19 non-infected controls. The decrease of two miRNAs, miR-451 and miR-16, was correlated not only with the infection of *Plasmodium* but also with its severity, defined on the basis of parasite densities [141]. However, the study was limited by the reduced number of patients and by the lack of a group of individuals infected with other pathogens to support that the differences observed in miRNAs were specific to the *Plasmodium* infection. Moreover, the down-regulation of miR-451, the most abundant miRNA in human erythrocytes, is surprising given that malaria infections usually generate high haemolysis and therefore release miRNAs from erythrocytes. Further studies are needed to explore the value of miRNAs as biomarkers of malaria infection.

## miRNAs as biomarkers in malaria disease

**Fig. 2:**
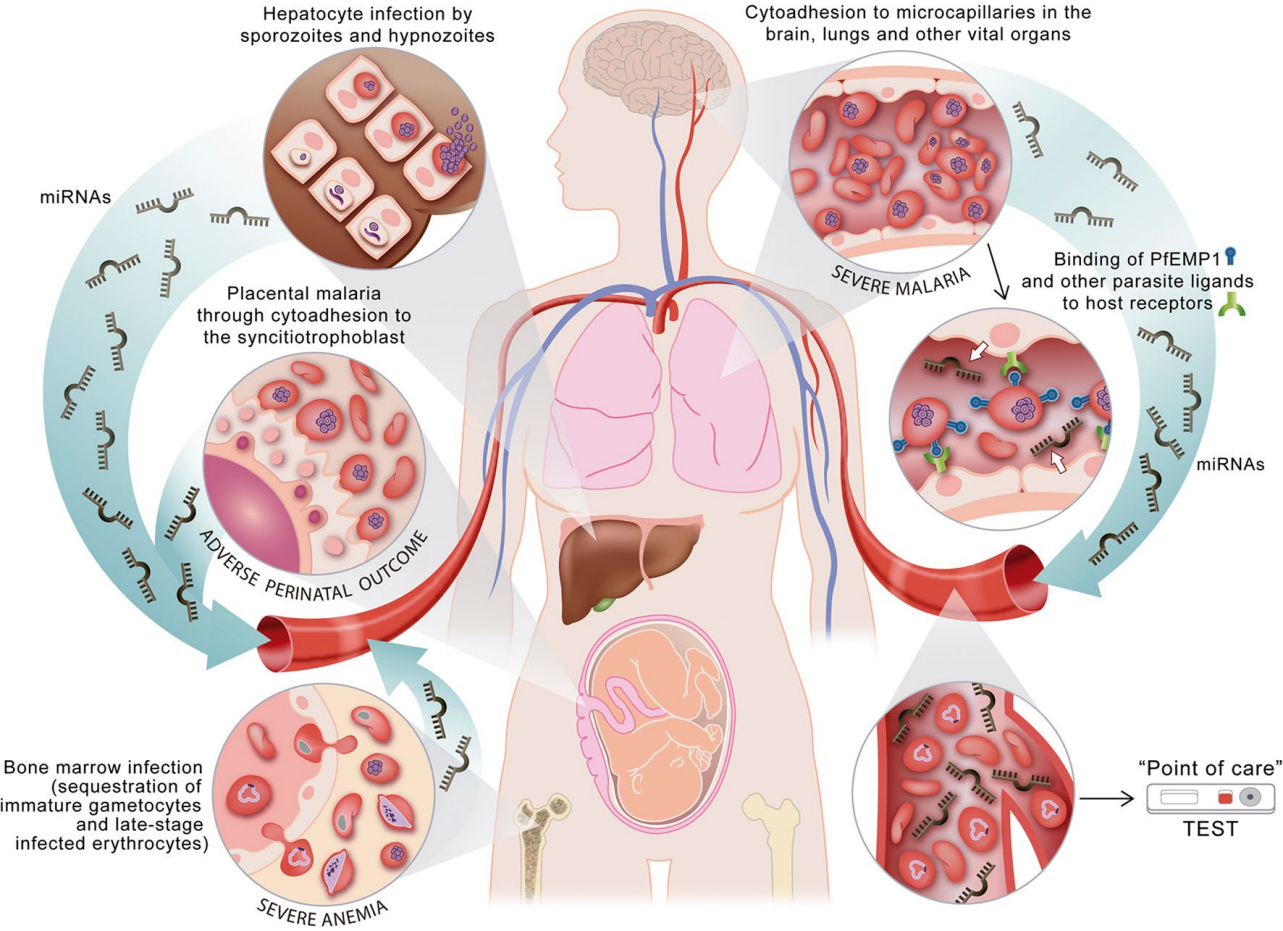
Potential links between human severe malaria physiopathology and miRNAs: a proposed model. Upon infection with *P. falciparum*, infected liver cells or tissues affected by parasite sequestration, such as the placenta, bone marrow or the brain, may produce tissue-specific miRNAs that are released to blood circulation. Detection of such miRNAs may allow discriminating between infected individuals with significant parasite sequestration and those in whom parasitaemia is unrelated to the cause of severity, and guide effective therapy. miRNAs might also be the basis for new diagnostic tools to predict malaria infections at risk of progression to severe disease, or of those asymptomatic infections that may progress to symptomatic malaria. Finally, miRNAs could be informative of the presence of parasites hidden in the liver (*P. vivax* or *P. ovale* hypnozoites)

The discovery of miRNAs has opened exciting possibilities to identify blood biomarkers of organ-specific injury. The content of miRNAs in the host is influenced by host-pathogen interactions, as has been shown for bacteria, viruses and apicomplexan parasites [137, 138, 142], including malaria parasites. Importantly, cyto-adhesion of *P. falciparum* to host receptors can trigger intracellular signals in target cells [143] and potentially affect a wide range of cellular responses regulated by miRNAs, such as the expression adhesion molecules [144], vascular inflammation and parenchymal damage of bone marrow cells [145]. These previous evidences suggest that *P. falciparum*, although not able to produce miRNAs, may manipulate host miRNAs, pointing to the potential of these small molecules to not only shed light on the molecular mechanisms involved in severe malaria and sickle cell resistance [133], but also to assess the degree of vital organ dysfunction associated with parasite sequestration, which is believed to constitute a key pathogenic event in *P. falciparum* [146]. Thus, miRNAs secreted by host tissues damaged by *P. falciparum* sequestration, for instance at the brain, lung, spleen or liver, might have the potential to be developed into a biomarker of malaria-specific severe disease (Fig. 2). If differentially expressed in severe malaria patients compared to uncomplicated malaria or to other severe pathologies, detection of sub-picomolar levels of miRNAs in blood [33] associated with *P. falciparum* sequestration may allow discriminating infected individuals with significant parasite sequestration from those in whom the parasitaemia is unrelated to the cause of severity [147]. Such an approach might allow the differentiation between true severe malaria from cases of incidental parasitaemia among individuals presenting at health centres with a severe infection. However, the fact that severe malaria can also result from an excessive host inflammatory response following exposure to malaria toxins imposes potential limitations to the link between all severe pathologies, parasite sequestration and production of tissue-specific miRNAs. Further studies are needed to understand if exposure to these malaria toxins can affect miRNA production by immune cell populations and be used to distinguish underlying mechanisms of severe malaria.

Composite diagnostics, including nucleic acid amplification of the pathogen and miRNA detection, to ascertain the pathological cause of the severe disease could thus guide effective therapy, ensuring that children receive prompt and correct treatment, and reducing the development of resistance. Such miRNAs can be also the basis for new diagnostic tools to predict malaria infections at risk of progression to severe disease that may require a more aggressive treatment. Moreover, miRNAs associated with malaria-related pathologies may also be applied to track the success of treatments against severe malaria cases, to generate estimates of malaria mortality rates, to evaluate outcomes in severe malaria clinical trials, and to improve measurements of the impact of future interventions against severe malaria. Finally, miRNAs could be also informative of the initial stages of liver infection, including hypnozoites in the case of *P. vivax* or *P. ovale* [71], hidden parasites in the bone marrow and its associated pathology eventually leading to severe anaemia [40], and of those asymptomatic infections that may progress to symptomatic malaria [17]. Finally, longitudinal assessment of miRNAs during the course of infections, including comparisons between asymptomatic and symptomatic individuals as well as severe malaria cases during treatment and after resolution, could allow the identification of miRNA signatures with value as a prognosis marker.

Robust and affordable point-of-care tests based on miRNA detection with laminar flow-assisted dendritic amplification on power-free microfluidic chip [148], or future field-deployable nucleic-acid based methods, would be useful for clinicians working in remote settings to improve their management of patients, as well as a more targeted allocation of resources at the health policy level. Current technologies do not allow the use of these devices in daily routine, especially in malaria-endemic countries. However, many efforts are being done to develop more affordable tools to detect miRNAs of diagnostic or prognostic value, as they can be potentially applied to different types of disease. New approaches are under development, such as detection methods based in fluorescence [149] that do not required previous amplification, or biosensors which can be used in portable devices [150]. Moreover, miRNA-based therapeutics based on anti-miR inhibitors or miRNA delivery are rapidly evolving, with some miRNAs currently starting their clinical development phase, such as MRX34, a liposome-encapsulated miR-34 mimic which inhibits multiple oncogenic pathways as well as stimulates anti-tumour immune response to induce cancer cell death [151]. Studies showing that transfection of one miRNA can contribute to increase the resistance to malaria in mice [140] suggest that miRNAs may be particularly interesting in seeking alternative therapeutic approaches given the dependence of apicomplexan over the host cellular machinery to accomplish infection and complete their biological cycles. Since miRNAs also have a role in modulating immune responses against invading parasites, these could be potential targets to enhance host responses against *P. falciparum* or to make host cells refractory to parasite cyto-adhesion. In addition, this knowledge can provide important clues about the physiopathological mechanisms involved in other infections and help to dissect the biogenesis and function of miRNAs in normal human cells.

## Conclusions

Although the enormous potential of miRNAs as a non-invasive biomarker for several human diseases is well known, little has been done to decipher their relationship with malaria pathogenesis. However, evidence is accumulating that host miRNA can be altered by parasite infections and be involved in the course and outcome of infectious diseases [142], pointing towards the potential of such small molecules as biomarkers for risk assessment, diagnosis, prognosis, and monitoring of treatments for malaria infections. The application of miRNAs in biofluids as a measure of tissue injury associated with parasite sequestration may overcome the main rate-limiting roadblock due to the impossibility of measuring the presence and distribution of parasites in host organs unless *post*-*mortem* samples can be obtained. This approach opens the exciting possibility of identifying new diagnostic targets for patients at risk of severe malaria, and to distinguish severe malaria cases from those associated with other common pathologies, and also to facilitate treatment decisions. Overall, this knowledge will open up new horizons and opportunities for research, not only on malaria and other infectious diseases, but also on the role of human miRNAs. Future studies will likely contribute to identify key blocking events that may be targeted to develop new effective methods for inhibiting or reversing the process of cyto-adhesion, as well as to develop miRNA-based biomarkers of diagnostic value for severe disease associated with *P. falciparum* sequestration.

## Abbreviations

AAV8: adeno-associated virus8
ANG1: angiopoietin 1
ANG2: angiopoietin 2
APC: activated protein C
circRNAs: circular RNA
CRP: C-reactive protein
EPCR: endothelial protein C receptor
EPO: erythropoietin
GAP: genetically attenuated parasites
HDL: high-density lipoprotein
HRP-2: histidine-rich protein 2
IP-10: IFN-gamma-inducible protein 10
IL-10: interleukin-10
IMCI: integrated management of childhood illness
LAMP: loop-mediated isothermal amplification
LDL: low-density lipoprotein
LDMS: laser desorption mass spectrometry
miRNA: microRNA
mRNA: messenger RNA
PCR: polymerase chain reaction
PCT: procalcitonin
PfEMP1: *Plasmodium falciparum* erythrocyte membrane protein 1
pre-miRNA: microRNA precursor
RDT: rapid diagnostic test
RISC: RNA-induced silencing complex
RNAi: RNA interference
siRNA: small interfering RNAs
TIMP-1: tissue inhibitor of metalloproteinases 1
TNF-α: tumour necrosis factor-α
TNFR2: tumour necrosis factor receptor 2
UTR: untranslated region
